# Exposure-Based Screening for Nipah Virus Encephalitis, Bangladesh

**DOI:** 10.3201/eid2102.141129

**Published:** 2015-02

**Authors:** Hossain M.S. Sazzad, Stephen P. Luby, Ute Ströher, Peter Daszak, Sharmin Sultana, Sayma Afroj, Mahmudur Rahman, Emily S. Gurley

**Affiliations:** icddr,b,^1^ Dhaka, Bangladesh (H.M.S. Sazzad, S. Afroj, E.S. Gurley);; Stanford University, Stanford, California, USA (S.P. Luby);; Centers for Disease Control and Prevention, Atlanta, Georgia, USA (U. Ströher);; EcoHealth Alliance, New York, New York, USA (P. Daszak);; Institute of Epidemiology, Disease Control and Research, Dhaka (S. Sultana, M. Rahman)

**Keywords:** Nipah virus, NiV, encephalitis, Nipah virus encephalitis, surveillance, screening, infection control, paramyxovirus, nosocomial infection, Bangladesh, viruses

## Abstract

We measured the performance of exposure screening questions to identify Nipah virus encephalitis in hospitalized encephalitis patients during the 2012–13 Nipah virus season in Bangladesh. The sensitivity (93%), specificity (82%), positive predictive value (37%), and negative predictive value (99%) results suggested that screening questions could more quickly identify persons with Nipah virus encephalitis.

Nipah virus (NiV) is a bat-borne paramyxovirus that causes encephalitis in humans and can be transmitted from person to person, posing a global pandemic threat (*1**–**4*). Because of the high costs of laboratory-based surveillance systems, maintaining surveillance for highly fatal but rare diseases, such as NiV encephalitis, is difficult for low-income countries, such as Bangladesh. In Bangladesh, NiV encephalitis outbreaks with a >70% case-fatality rate have occurred almost every year since 2001 during December–April (*3**–**5*). Where NiV encephalitis outbreaks had been repeatedly identified, the Institute for Epidemiology Disease Control and Research of the Government of Bangladesh, in collaboration with icddr,b, started hospital-based encephalitis surveillance in February 2006.

Before illness onset, nearly every NiV encephalitis case-patient identified in Bangladesh was exposed either to raw or fermented date palm sap or to another NiV encephalitis case-patient (*3**–**6*). Bangladeshis collect and drink raw date palm sap fresh during December–April (*3**–**5*). *Pteropus* fruit bats come in contact with date palm sap by landing on the sap stream and contaminate sap with saliva and/or urine (*7**,**8*). NiV RNA has been identified in human blood, urine, respiratory secretions, and saliva (*4**,**9*). Epidemiologic studies indicate that respiratory secretions and saliva are the most likely vehicles of NiV transmission from patients to caregivers (*3**,**4*). Therefore, implementation of standard, contact, and droplet precautions (*10*) could reduce NiV transmission in hospitals. However, hospitals with few resources for infection control are unable to implement standard, contact, and droplet precautions for all patients with encephalitis (*11*), which places caregivers at risk for person-to-person transmission of NiV (*4**,**12*).

Because no rapid diagnostic tests for NiV infection are currently available, surveillance for NiV encephalitis in Bangladesh relies on a central laboratory in the capital (Dhaka) to confirm diagnosis several days or weeks after sample collection. Earlier identification of NiV encephalitis cases would enable targeted infection control efforts to reduce the number of persons exposed to NiV-infected patients and thereby reduce subsequent secondary transmission of NiV from person to person. The objective of this study was to compare the performance of screening questions about recent exposure to date palm sap or other encephalitis patients with serologic testing results to more quickly identify NiV encephalitis cases in our surveillance hospitals.

## The Study

Since 2006, surveillance physicians have listed and collected blood from those patients encephalitis, defined as fever or history of fever with axillary temperature >38.5°C (101.3°F) and altered mental status, new onset of seizures, or new neurologic deficit—in patients admitted to 3 Nipah surveillance hospitals: Rajshahi, Rangpur, and Faridpur Medical College hospitals. The Institute for Epidemiology Disease Control and Research and US Centers for Disease Control and Prevention tested serum with an IgM-capture enzyme immunoassay to detect NiV IgM, and we defined laboratory-confirmed NiV encephalitis as NiV IgM in serum.

During December 2012–March 2013, surveillance physicians interviewed accompanying caregivers of all hospitalized patients whose illness met the encephalitis case definition on admission in the inpatient ward. Study physicians asked about patients’ consumption of raw or fermented date palm sap and contact with other persons with fever and altered mental status in the month before illness onset; if caregivers were unaware of the patient’s exposures, study physicians asked them phone the patient’s friends and colleagues about exposures. Exposures were recorded in surveillance log books. Hospital physicians used personal protection equipment and provided it to caregivers of each patient with encephalitis and a history of these exposures.

As part of subsequent epidemiologic studies, we also conducted detailed case investigations at each NiV encephalitis case-patient’s household. We interviewed surviving patients directly, or appropriate proxies among family, friends and relatives for patients who died, about their exposures to encephalitis patients or to fresh or fermented date palm sap before illness.

We calculated the sensitivity, specificity, positive predictive value (PPV), and negative predictive value (NPV) of the screening questions asked on admissions to hospitals by comparing with the NiV IgM results. We repeated the calculations for patients hospitalized during January and February, when the prevalence of NiV encephalitis is highest. We compared the answers provided by caregivers during patient hospitalization with those provided during interviews in the community as part of our epidemiologic studies. icddr,b’s Ethical Review Committee reviewed and approved the protocol for NiV surveillance and case investigation.

Surveillance physicians identified 360 patients with encephalitis during December 2012–March 2013. They collected and tested blood samples from 328 (91%) patients for NiV IgM. Seventeen (5%) had NiV IgM (Table 1), of whom 15 (88%) NiV encephalitis case-patients were identified during January and February 2013. Of the 17 confirmed case-patients, family caregivers of 14 reported either a history of drinking raw or fermented date palm sap or contact with other persons with fever and altered mental status in the month before illness onset. Therefore, the sensitivity of the screening questions was 82%, specificity was 86%, PPV was 24% and NPV was 99% (Table 2). The sensitivity during January–February was 93%, specificity was 82%, PPV was 37%, and NPV was 99%.

**Table 1:** Proportion of encephalitis patients with history of reported exposures to drinking date palm sap or contact with patients with encephalitis by Nipah serodiagnosis in 3 surveillance hospitals, Bangladesh, 2012–13 Nipah virus season

Reported risk factor	Overall, Dec–Mar, no. (%)				Peak incidence during Jan–Feb, no. (%)		
IgM positive, n = 17	IgM negative, n = 311	Total, n = 328		IgM positive, n = 15	IgM negative, n = 132	Total, n = 147
History of drinking raw date palm sap	9 (47)	45 (14)	54 (16)		9 (60)	24 (18)	33 (22)
History of drinking fermented date palm sap	2 (11)	0	2 (1)		2 (13)	0	2 (1)
History of contact with other encephalitis patients	3 (18)	0	3 (1)		3 (20)	0	3 (2)
No history of drinking raw or fermented date palm sap or contact with encephalitis patients	3 (18)	266 (86)	269 (82)		1 (7)	108 (5)	109 (47)

**Table 2:** Comparison of results of screening for exposure to* and results of serologic testing for Nipah virus encephalitis among patients with encephalitis at 3 surveillance hospitals, Bangladesh, December 2012–March 2013

Months	Prevalence (95% CI), %	Sensitivity (95% CI), %	Specificity (95% CI), %	Positive predictive value (95% CI), %	Negative predictive value (95% CI), %
Dec 2012–Mar 2013	5 (3–8)	82 (57–96)	86 (81–89)	24 (14–37)	99 (97–100)
Jan–Feb 2013	10 (6–16)	93 (68–100)	82 (74–88)	37 (22–54)	99 (95–100)

*Drinking raw or fermented date palm sap or having contact with encephalitis patients in month before illness onset.

At admission, 3 (18%) NiV encephalitis case-patients had no reported history of drinking raw or fermented date palm sap or of contact with persons who had encephalitis (Table 1). However, during the epidemiologic investigations in the community, family members of 2 case-patients reported that the patients drank fermented date palm sap in the month before illness onset. Of the 14 NiV encephalitis case-patients who, at admission, had reported 1 of the risk exposures, results were consistent with exposures reported during the epidemiologic investigation.

## Conclusions

Screening patients with possible encephalitis at the time they seek hospital care regarding recent exposure to date palm sap and to other patients with encephalitis demonstrated high sensitivity and specificity for detecting NiV encephalitis, particularly during peak months of NiV encephalitis incidence. The high NPV of the screening questions suggests that focusing infection control efforts toward patients with these exposures is an efficient use of scarce resources to prevent transmission. Although three fourths of encephalitis patients had reported histories of exposure, they possibly could have had other infections, including other bat-borne viruses, that were transmitted through similar routes or could have lacked NiV IgM, despite having NiV infection (*13*). Alternatively, recent consumption of date palm sap by these patients might have been purely coincidental because this practice is common in Bangladesh during this season, but Nipah infection is rare.

For 2 NiV encephalitis case-patients, caregivers did not report a history of drinking fermented date palm sap during hospital interview, but this behavior was reported in later community investigations. Because 90% of Bangladeshis are Muslim, and consumption of alcohol is prohibited by Islam (*14*) and illegal in Bangladesh, patients might be reluctant to report drinking traditional liquor made of fermented date palm sap. Therefore, caregivers should be asked about socially stigmatized behaviors privately and confidentially to increase the odds that these stigmatized behaviors are reported.

Exposure-based screening can detect patients at high risk for NiV encephalitis in low-income, resource-constrained settings, such as Bangladesh. We deployed screening questions on admission to inpatient wards but screening earlier, at triage in emergency wards, could further reduce risk. Surveillance for other diseases with well-described exposures that put healthcare workers at risk, such as Ebola virus infection, and where laboratory diagnosis is limited or delayed could also deploy this approach.
